# Supplementary material for: Forecasting drug resistant HIV protease evolution
Source: bioRxiv. 2025 Apr 4:2025.03.31.646462. Preprint. [Version 1] doi: 10.1101/2025.03.31.646462 (PMC12190175; doi:10.1101/2025.03.31.646462)
Supplement: Supplement 1 [file media-1.pdf]

Table 1

Treatment regimen	#seqs			
None	40820			
IDV	558			
SQV	155			
NFV	514			
RTV	150			
IDV,RTV,SQV	153			
RTV,SQV	107			
IDV,NFV,RTV,SQV	179			
IDV,NFV	166			
LPV	1379			
ATV,RTV	185			

Position	Unique amino acids (AA) observed	#AA
1	['A', 'F', 'H', 'L', 'P', 'Q', 'R', 'S', 'T']	9
2	['*', 'E', 'H', 'I', 'K', 'L', 'P', 'Q', 'R', 'S', 'T', 'V']	12
3	['F', 'I', 'K', 'L', 'M', 'N', 'Q', 'S', 'T', 'V']	10
4	['A', 'C', 'D', 'F', 'H', 'I', 'K', 'L', 'N', 'P', 'Q', 'S', 'T']	13
5	['A', 'F', 'H', 'I', 'L', 'P', 'Q', 'R', 'S', 'T', 'V']	11
6	['*', 'C', 'E', 'G', 'L', 'P', 'R', 'S', 'W']	9
7	['*', 'D', 'E', 'H', 'K', 'L', 'P', 'Q', 'R', 'S', 'T', 'Y']	12
8	['*', 'E', 'G', 'H', 'L', 'P', 'Q', 'R', 'W']	9
9	['A', 'G', 'H', 'L', 'P', 'Q', 'S', 'T']	8
10	['A', 'C', 'F', 'G', 'H', 'I', 'L', 'M', 'N', 'P', 'Q', 'R', 'S', 'T', 'V', 'Y']	16
11	['A', 'C', 'D', 'F', 'G', 'I', 'K', 'L', 'M', 'S', 'T', 'V']	12
12	['*', 'A', 'D', 'E', 'F', 'G', 'H', 'I', 'K', 'L', 'M', 'N', 'P', 'Q', 'R', 'S', 'T',	20
13	['A', 'C', 'D', 'E', 'F', 'G', 'I', 'K', 'L', 'M', 'N', 'R', 'T', 'V', 'Y']	15
14	['A', 'D', 'E', 'G', 'H', 'I', 'K', 'L', 'M', 'N', 'Q', 'R', 'S', 'T', 'V', 'W', 'Y'	17
15	['*', 'A', 'D', 'E', 'G', 'I', 'K', 'L', 'M', 'N', 'R', 'S', 'T', 'V', 'Y']	15
16	['A', 'C', 'D', 'E', 'G', 'K', 'N', 'Q', 'R', 'V', 'W', 'Y']	12
17	['A', 'C', 'D', 'E', 'G', 'K', 'N', 'R', 'S', 'T', 'V']	11
18	['*', 'A', 'E', 'G', 'H', 'I', 'K', 'L', 'M', 'N', 'P', 'Q', 'R', 'S', 'T', 'V', 'Y']	17
19	['A', 'E', 'F', 'G', 'H', 'I', 'K', 'L', 'M', 'N', 'P', 'Q', 'R', 'S', 'T', 'V', 'W'	17
20	['*', 'C', 'E', 'G', 'I', 'K', 'L', 'M', 'N', 'Q', 'R', 'T', 'V', 'Y']	14
21	['A', 'D', 'E', 'G', 'K', 'N', 'Q', 'R', 'T', 'V']	10
22	['A', 'C', 'D', 'G', 'L', 'P', 'R', 'S', 'T', 'V']	10
23	['F', 'H', 'I', 'K', 'L', 'P', 'Q', 'S', 'V']	9
24	['*', 'F', 'I', 'L', 'M', 'P', 'S', 'V', 'W']	9
25	['D', 'E', 'F', 'G', 'H', 'K', 'N', 'V', 'Y']	9
26	['A', 'I', 'P', 'R', 'S', 'T']	6
27	['E', 'G', 'P', 'R']	4
28	['A', 'E', 'G', 'P', 'Q', 'T', 'V']	7
29	['A', 'D', 'E', 'G', 'H', 'N', 'V', 'Y']	8
30	['D', 'E', 'G', 'K', 'N', 'S', 'V', 'Y']	8
31	['A', 'G', 'I', 'P', 'R', 'S', 'T']	7
32	['A', 'E', 'F', 'I', 'L', 'M', 'T', 'V']	8
33	['F', 'I', 'K', 'L', 'M', 'S', 'T', 'V']	8
34	['*', 'A', 'D', 'E', 'G', 'H', 'K', 'L', 'N', 'Q', 'S', 'T', 'V']	13
35	['*', 'A', 'D', 'E', 'G', 'H', 'K', 'N', 'Q', 'R', 'S', 'T', 'V', 'Y']	14
36	['A', 'D', 'E', 'F', 'I', 'K', 'L', 'M', 'N', 'S', 'T', 'V']	12
37	['A', 'C', 'D', 'E', 'F', 'G', 'H', 'I', 'K', 'L', 'M', 'N', 'P', 'Q', 'R', 'S', 'T',	20
38	['C', 'F', 'G', 'I', 'K', 'L', 'M', 'S', 'V', 'W']	10
39	['A', 'E', 'H', 'I', 'K', 'L', 'M', 'P', 'Q', 'R', 'S', 'T', 'V']	13

40	['E', 'G', 'R', 'T']	4
41	['*', 'A', 'D', 'E', 'G', 'H', 'I', 'K', 'N', 'P', 'Q', 'R', 'S', 'T', 'V', 'Y']	16
42	['*', 'C', 'G', 'L', 'M', 'R', 'W', 'Y']	8
43	['*', 'A', 'E', 'G', 'I', 'K', 'M', 'N', 'Q', 'R', 'S', 'T', 'V']	13
44	['A', 'K', 'L', 'P', 'Q', 'R', 'S', 'T']	8
45	['*', 'I', 'K', 'L', 'M', 'N', 'P', 'Q', 'R', 'T', 'V']	11
46	['I', 'K', 'L', 'M', 'R', 'T', 'V']	7
47	['A', 'I', 'K', 'L', 'M', 'R', 'T', 'V']	8
48	['A', 'E', 'G', 'I', 'K', 'L', 'M', 'Q', 'R', 'T', 'V', 'W']	12
49	['A', 'E', 'G', 'K', 'R', 'V']	6
50	['F', 'I', 'L', 'M', 'N', 'S', 'V']	7
51	['A', 'E', 'G', 'K', 'R', 'V', 'W']	7
52	['A', 'D', 'E', 'F', 'G', 'R', 'S', 'V']	8
53	['C', 'F', 'I', 'L', 'S', 'V', 'W', 'Y']	8
54	['A', 'F', 'I', 'K', 'L', 'M', 'R', 'S', 'T', 'V']	10
55	['E', 'F', 'G', 'H', 'I', 'K', 'M', 'N', 'Q', 'R', 'T']	11
56	['A', 'G', 'I', 'K', 'L', 'R', 'T', 'V']	8
57	['*', 'G', 'I', 'K', 'N', 'Q', 'R', 'S', 'T']	9
58	['*', 'E', 'H', 'K', 'L', 'P', 'Q', 'R']	8
59	['C', 'D', 'F', 'H', 'M', 'N', 'Q', 'S', 'Y']	9
60	['A', 'D', 'E', 'G', 'H', 'K', 'N', 'R', 'S', 'T', 'Y']	11
61	['A', 'D', 'E', 'G', 'H', 'K', 'L', 'N', 'P', 'Q', 'R', 'S', 'Y']	13
62	['G', 'I', 'K', 'L', 'M', 'N', 'Q', 'R', 'T', 'V']	10
63	['A', 'C', 'D', 'E', 'F', 'G', 'H', 'I', 'K', 'L', 'M', 'N', 'P', 'Q', 'R', 'S', 'T',	20
64	['F', 'I', 'K', 'L', 'M', 'P', 'R', 'T', 'V']	9
65	['A', 'D', 'E', 'G', 'H', 'I', 'K', 'N', 'Q', 'R', 'T', 'V', 'Y']	13
66	['C', 'E', 'F', 'I', 'L', 'M', 'N', 'S', 'T', 'V']	10
67	['*', 'A', 'C', 'D', 'E', 'F', 'G', 'H', 'K', 'L', 'M', 'N', 'Q', 'R', 'S', 'W', 'Y	17
68	['D', 'E', 'G', 'K', 'Q', 'R', 'V', 'W']	8
69	['A', 'C', 'E', 'H', 'I', 'K', 'L', 'M', 'N', 'P', 'Q', 'R', 'S', 'T', 'Y']	15
70	['A', 'E', 'G', 'H', 'I', 'K', 'L', 'M', 'N', 'P', 'Q', 'R', 'S', 'T', 'V', 'Y']	16
71	['A', 'D', 'G', 'I', 'L', 'M', 'N', 'P', 'S', 'T', 'V']	11
72	['A', 'D', 'E', 'F', 'I', 'K', 'L', 'M', 'N', 'P', 'Q', 'R', 'S', 'T', 'V', 'W', 'Y']	17
73	['A', 'C', 'D', 'G', 'K', 'N', 'R', 'S', 'T', 'V']	10
74	['A', 'E', 'I', 'K', 'M', 'P', 'Q', 'R', 'S', 'T', 'V']	11
75	['A', 'G', 'I', 'L', 'M', 'V']	6
76	['E', 'F', 'I', 'L', 'S', 'V']	6
77	['A', 'E', 'G', 'I', 'K', 'L', 'M', 'Q', 'T', 'V']	10
78	['*', 'A', 'D', 'E', 'G', 'R', 'V', 'Y']	8
79	['A', 'D', 'E', 'H', 'L', 'N', 'P', 'Q', 'S', 'T']	10

80	['A', 'H', 'I', 'P', 'Q', 'R', 'S', 'T', 'V']	9
81	['H', 'L', 'P', 'S', 'T']	5
82	['A', 'C', 'D', 'E', 'F', 'H', 'I', 'L', 'M', 'N', 'P', 'S', 'T', 'V']	14
83	['D', 'H', 'I', 'K', 'N', 'P', 'S', 'T', 'Y']	9
84	['A', 'C', 'I', 'K', 'L', 'M', 'T', 'V']	8
85	['F', 'I', 'L', 'M', 'N', 'T', 'V']	7
86	['*', 'C', 'E', 'G', 'K', 'R', 'V']	7
87	['*', 'E', 'G', 'I', 'K', 'P', 'Q', 'R', 'S', 'T', 'V']	11
88	['D', 'G', 'H', 'I', 'K', 'N', 'S', 'T', 'V', 'Y']	10
89	['A', 'C', 'E', 'F', 'I', 'K', 'L', 'M', 'P', 'R', 'T', 'V', 'W']	13
90	['*', 'C', 'F', 'L', 'M', 'S', 'V', 'W']	8
91	['A', 'C', 'G', 'I', 'K', 'L', 'N', 'P', 'S', 'T', 'V']	11
92	['*', 'A', 'E', 'G', 'H', 'K', 'L', 'M', 'N', 'P', 'Q', 'R', 'S', 'T']	14
93	['A', 'D', 'F', 'H', 'I', 'K', 'L', 'M', 'N', 'P', 'R', 'S', 'T', 'V', 'Y']	15
94	['A', 'C', 'D', 'E', 'G', 'H', 'N', 'Q', 'R', 'S', 'V', 'W']	12
95	['A', 'C', 'F', 'G', 'I', 'L', 'M', 'N', 'R', 'S', 'V', 'W', 'Y']	13
96	['A', 'D', 'I', 'L', 'N', 'P', 'S', 'T']	8
97	['*', 'F', 'I', 'K', 'L', 'P', 'Q', 'S', 'T', 'V']	10
98	['C', 'D', 'H', 'I', 'K', 'N', 'Q', 'R', 'S', 'T', 'Y']	11
99	['F', 'G', 'I', 'L', 'S', 'V', 'W', 'Y']	8

